# Autism-associated gene *shank3* is necessary for social contagion in zebrafish

**DOI:** 10.1186/s13229-023-00555-4

**Published:** 2023-06-30

**Authors:** Kyriacos Kareklas, Magda C. Teles, Elena Dreosti, Rui F. Oliveira

**Affiliations:** 1 Instituto Gulbenkian de Ciência, R. Q.ta Grande 6, 2780-156 Oeiras, Portugal; 2 ISPA - Instituto Universitário, Rua Jardim do Tabaco 34, 1149-041 Lisbon, Portugal; 3 Department of Cell and Developmental Biology, University College London, London, UK

**Keywords:** Autism, SHANK3, Social Contagion, Zebrafish, Affect, Recognition, Attention, Neuroplasticity, Synaptogenesis, Neuroligins

## Abstract

**Background:**

Animal models enable targeting autism-associated genes, such as the *shank3* gene, to assess their impact on behavioural phenotypes. However, this is often limited to simple behaviours relevant for social interaction. Social contagion is a complex phenotype forming the basis of human empathic behaviour and involves attention to the behaviour of others for recognizing and sharing their emotional or affective state. Thus, it is a form of social communication, which constitutes the most common developmental impairment across autism spectrum disorders (ASD).

**Methods:**

Here we describe the development of a zebrafish model that identifies the neurocognitive mechanisms by which *shank3* mutation drives deficits in social contagion. We used a CRISPR-Cas9 technique to generate mutations to the *shank3a* gene, a zebrafish paralogue found to present greater orthology and functional conservation relative to the human gene. Mutants were first compared to wild types during a two-phase protocol that involves the observation of two conflicting states, distress and neutral, and the later recall and discrimination of others when no longer presenting such differences. Then, the whole-brain expression of different neuroplasticity markers was compared between genotypes and their contribution to cluster-specific phenotypic variation was assessed.

**Results:**

The *shank3* mutation markedly reduced social contagion via deficits in attention contributing to difficulties in recognising affective states. Also, the mutation changed the expression of neuronal plasticity genes. However, only downregulated neuroligins clustered with *shank3a* expression under a combined synaptogenesis component that contributed specifically to variation in attention.

**Limitations:**

While zebrafish are extremely useful in identifying the role of *shank3* mutations to composite social behaviour, they are unlikely to represent the full complexity of socio-cognitive and communication deficits presented by human ASD pathology. Moreover, zebrafish cannot represent the scaling up of these deficits to higher-order empathic and prosocial phenotypes seen in humans.

**Conclusions:**

We demonstrate a causal link between the zebrafish orthologue of an ASD-associated gene and the attentional control of affect recognition and consequent social contagion. This models autistic affect-communication pathology in zebrafish and reveals a genetic attention-deficit mechanism, addressing the ongoing debate for such mechanisms accounting for emotion recognition difficulties in autistic individuals.

**Supplementary Information:**

The online version contains supplementary material available at 10.1186/s13229-023-00555-4.

## Background

Social bonds are strengthened by the communication of emotions or affective states, such as fear or distress, between individuals [1–3]. This manifests in the phenomenon of social contagion, which is evolutionarily conserved, and it consists in the transmission of affective states to others and constitutes the basis for more complex prosocial or empathic behaviour in humans, such as consolation and concern [3–5]. Social contagion relies on socio-cognitive and social communication traits, such as the ability to behaviourally express emotions, as well as to perceive, recognise, and share those of others, which exhibit deficits in humans with autism spectrum disorders (ASD) [6–9]. These deficits can be the result of certain genetic syndromes that emphasise the social pathologies of ASD. A prominent example is the Phelan-McDermid syndrome, which is the result of deletions to the long arm end of chromosome 22 [10–12]. Among these deletions, those to the gene for the protein SHANK3 have been particularly linked to ASD. The protein belongs to a set of postsynaptic density multidomain scaffold proteins connecting membrane proteins to the actin cytoskeleton and to G-protein-coupled signalling pathways, including neurotransmitter receptors and ion channels, and contributing to synapse formation and dendritic spine maturation.

Animal models targeting candidate autism genes are typically linked to generic autistic pathology, limited to simple behaviours of social interaction and rarely extending to complex phenotypes, such as social contagion. For example, deletions to the mice and rat *shank3* gene elicit deficiencies in inter-individual communication, particularly social vocalizations, simple social recognition, such as discrimination between familiar from unfamiliar others, and prosocial behaviour, such as allogrooming [12, 13]. Social contagion is a composite of these abilities, defined by the communication of behavioural signals, the recognition of underlying affective states in others, as a requisite upstream process, and often resulting in downstream other-oriented or prosocial responses [14, 15]. In humans, ASD-derived deficiencies in empathy and difficulties in recognising emotions in others, such as fear or distress [6–9], have been related to altered activity in key social decision-making brain areas, particularly the amygdala, which can be recovered by intranasal oxytocin treatments [16, 17]. Similarly, distress recognition in rodents has been demonstrated to rely on conserved oxytocinergic controls in the amygdala that regulate the discrimination of affective from neutral behaviour in others, while this also drives consequent approaches towards those in distress [18]. Together, this evidence suggests that ASD-like pathology across evolutionarily distant species may include social recognition and contagion impairments that underlie ASD empathic deficits in humans, and that *shank3* mutations could be a conserved causal factor.

**Fig. 1:**
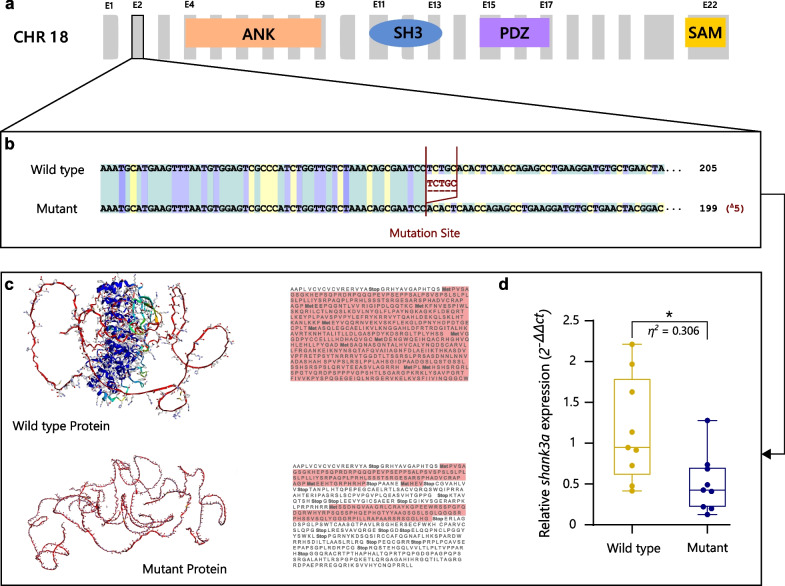
Characterization of the genetic mutation of *shank3a* and its effects on protein transcription and *shank3a* expression. (**a**) The *shank3a* paralogue is found on chromosome 18, where mutation to Exon 2 precedes the localisation of the four typical SHANK3 domains, ankyrin repeats (ANK), SRC Homology 3 (SH3), PDZ and sterile α motif (SAM). (**b**) The genetic mutation induced in our study included a five base pair deletion that elicits (**c**) transcription of a markedly truncated protein structure and (**d**) significant decreases in *shank3a* expression

Recently, we demonstrated that the social contagion of alarm-induced distress behaviour in zebrafish, a well-characterised repertoire of erratic swimming and freezing, is dependent on its discrimination from neutral swimming states in others and on putatively homologous oxytocin-controlled mechanisms [19]. Furthermore, mutations to the *shank3* gene in zebrafish have separately been associated with brain development, motor skill and social interaction deficits similar to those observed in humans with ASD [20]. These conserved functions of the *shank3* gene are coupled with a highly conserved structure, where amino acid identity between human and zebrafish orthologs is up to 83.3 – 84.5% by some estimates, and at least 55–59% for the longest zebrafish *shank3* protein, and ≥ 80% when comparing the four archetypal *shank3* domains (ANK, SH3, PDZ, and SAM; Fig. 1a) [21, 22]. Furthermore, orthologs in other vertebrate species, including humans, other primates, and a set of key model species such as rats, mice, zebra finches and xenopus frogs, exhibit low genetic distances of less than 0.3 in relative number of nucleotide substitutions [21]. Together the evidence suggests that *shank3* mutations in zebrafish may elicit deficiencies in conserved social functions related to ASD pathology, including the recognition and transmission of affective states during social contagion.

Contrary to humans, the *shank3* gene is duplicated in zebrafish, with developmental expression patterns of the *shank3a* and the *shank3b* paralogues ranging from 2 to 120 h post fertilization [22]. Although six *shank3* transcripts have been described thus far, four of these are *shank3a* variants that display higher expression levels than the *shank3b* transcripts during development, particularly in the period immediately preceding the onset of zebrafish social competence development (i.e. 1- 5 days post fertilization) [21–23]. In addition, human SHANK3 protein–protein interaction motifs are more highly conserved in *shank3a* than *shank3b*, which suggests that *shank3a* is more suitable for translational models [22]. However, the modelling of ASD pathology in zebrafish is still in its infancy and the link between the *shank3* gene and social contagion, as well as underlying attention and recognition components, has yet to be made in any non-human model species. Here we address this gap by using a zebrafish *shank3a* mutant (Fig. 1) to test the conserved contribution of the *shank3* protein to the basal abilities underlying the evolution of emotion recognition and empathy and to model ASD pathology related to these abilities in humans.

Specific associations of *shank3* mutations to human and rodent-modelled ASD phenotypes particularly include deficits in verbal and non-verbal communication, which are related to mutations to the *shank3* domains conserved in zebrafish, particularly ANK and SH3 [24–27]. However, the well-established mechanistic role of *shank3* deletions to overall ASD pathology [28–31] is expected to extent phenotypic deficits to social contagion, which includes low attentiveness to behavioural cues in others, such as facial expressions [32, 33], and difficulties in recognizing the underlying affective states signalled by these cues, such as distress [6–9]. Attention to behavioural cues in others constitutes the basis upon which the ability to communicate affective signals relies and is a requisite for the recognition and consequent social transmission of affective states [32–35]. Moreover, ASD deficits in attention and social communication have been linked to altered neuroplasticity, including changes in synaptogenesis (e.g. postsynaptic *shank3* interactions with transmembrane neuroligin-neurexin function [27, 36, 37]), synaptic strengthening [38–40] and neurogenesis (differentiation and proliferation [41, 42]). Therefore, we hypothesise that *shank3* mutant zebrafish exhibit: (1) low social contagion of affective states, such as distress, due to (2) difficulties in recognising them, and that this is a cascade effect stemming from (3) attentional deficits (4) related to changes in neuroplasticity. To test this, we used an established two-stage video-playback social contagion protocol [19] and carried out a whole-brain characterization of genetic neuroplasticity markers that have been previously validated by our group [43]. During the social contagion tests, wild type and *shank3a* mutant animals first observed two simultaneous videos of a conspecific demonstrator, one exhibiting distress and the other one maintaining a neutral state, where attentional shifts and contagion could be measured. Then, at the second stage, animals were allowed to approach each video-image while both presented the demonstrator in a neutral state and where local preferences indicated whether the previously observed distinct states were recognised, encoded, and used to organise a motivated avoidance or approach. Finally, we used quantitative real-time PCR (qPCR) to measure relative expression levels of neuroplasticity genes, including neuroligins (*nlgn1, nlgn2*), which are involved in synaptogenesis; *bdnf*, which is implicated in synaptic strength; *npas4*, involved in GABAergic synapse establishment; *wnt3*, involved in neuronal proliferation*;* and *neurod,* which regulates neuronal differentiation [27, 36–43]. With this, we were able to develop a translational model for quantifying social-contagion deficits imposed by a candidate gene for ASD and to identify the cognitive and associated neuroplasticity components involved in these deficits.

## Methods

The aim of this study was to model in zebrafish the effects of *shank3* genetic mutations on the social contagion of an affective state, to identify underlying attentional and affective-sate recognition mechanisms, and to quantify associated changes in neuroplasticity at the gene expression level.

### Animals and husbandry

We used naïve adult zebrafish, *Danio rerio*, aged 6–12 months of the genetically modified *shank3a* mutant line (n = 12) and its wild-type background siblings as controls (n = 11). Fish were raised under laboratory conditions in the fish facility of the Gulbenkian Institute of Science, housed in groups of 11 fish in 1.5 L aquaria of a recirculating system (ZebraTec, 93 Tecniplast) kept at 27–28^O^C, 7.5 ± 0.2 pH, 1000 μSm conductivity and 14 L: 10 D photoperiod. Animals were fed a combination of live (*Artemia salina*) and dry food (*Gemma*). Welfare and health-maintenance protocols included a previously described approach where animals were kept with minimal external stress, full social and environmental enrichment, regular observational body condition and health checks, and free from known pathogens via sentinel testing.

### Genotypic characterization of shank3 mutants

To generate mutants for *shank3a*, the following Crispr target sequence for exon2 was identified using the ChopChop CRISPR design tool (chopchop.cbu.uib.no), GGCTCTGGTTGAGTGTGCAG. The generation of the sgRNA guide was obtained by using the technique described by Gagnon et al. [44]. The following DNA sequence was ordered from Invitrogen, TAATACGACTCACTATAGGCTCTGGTTGAGTGTG CAGGTTTTAGAGCTAGAAATAGCAAGTTAAAATAAGGC TAGTCCGT TATCAACTTG AAAAAGTGGCACCGAGTCGGTGCTTTTAAA, and annealed to the complementary oligo as described in the paper by Gagnon et al. The gRNA was generated using the HiScribe T7 High Yield RNA Synthesis Kit (NEB) followed by DNase I (NEB) digestion and purification with RNeasy MiniKit (Qiagen). The Cas9 from plasmid pT3TS-nCas9n (Addgene) was linearised with XbaI (NEB) and capped mRNA generated with the mMessage mMachine T3 Transcription Kit (Life Technologies) followed by polyadenylation with the Poly(A) Tailing Kit (Life Technologies). The synthesised mRNA was purified using the RNeasy MiniKit. Cas9 mRNA and *shank3a* gRNA were co-injected into one-cell stage embryos at a concentration of 200 ng and 75 ng per embryo, respectively.

Genomic DNA was extracted from single injected embryos by incubation in 50 μl base solution (1.25 M KOH and 10 mM EDTA) at 95 °C for 30 min followed by addition of 50 μl neutralization solution (2 M Tris HCL). To identify mutants, a region of 291 bp around the CRISPR target site was PCR-amplified with Taq DNA polymerase (Invitrogen) using primers Fwd 5′- GCT CTG GTG ACT TTG GTT GA -3′ and Rev 5′- CCT TCA CAC AGG TCA GAG AAG -3′. A frameshift mutation with a deletion of 5 bp in exon2 was identified (sequence CGAATC**CTCTG**CACACTCAACCA; deletion shown in bold), leading to compromised downstream functional domains according to gene structure (Ensembl ID: ENSDARG00000063332; NCBI ID: 557,701) and resulting to severe changes to amino-acid sequence and consequent alterations in protein structure compared to the wild-type protein (Fig. 1a–c). To confirm the expected non-sense mediated decay effects of this frameshift mutation, we ran whole-brain qPCR analyses in a sub-sample of our experimental animals and calculated relative expression levels of *shank3* RNA, which were found significantly reduced in mutant animals compared to wild types (*t *_*16*_ = 2.66, *P* = 0.0172; Fig. 1d).

### Experimental set-up and procedure

**Fig. 2:**
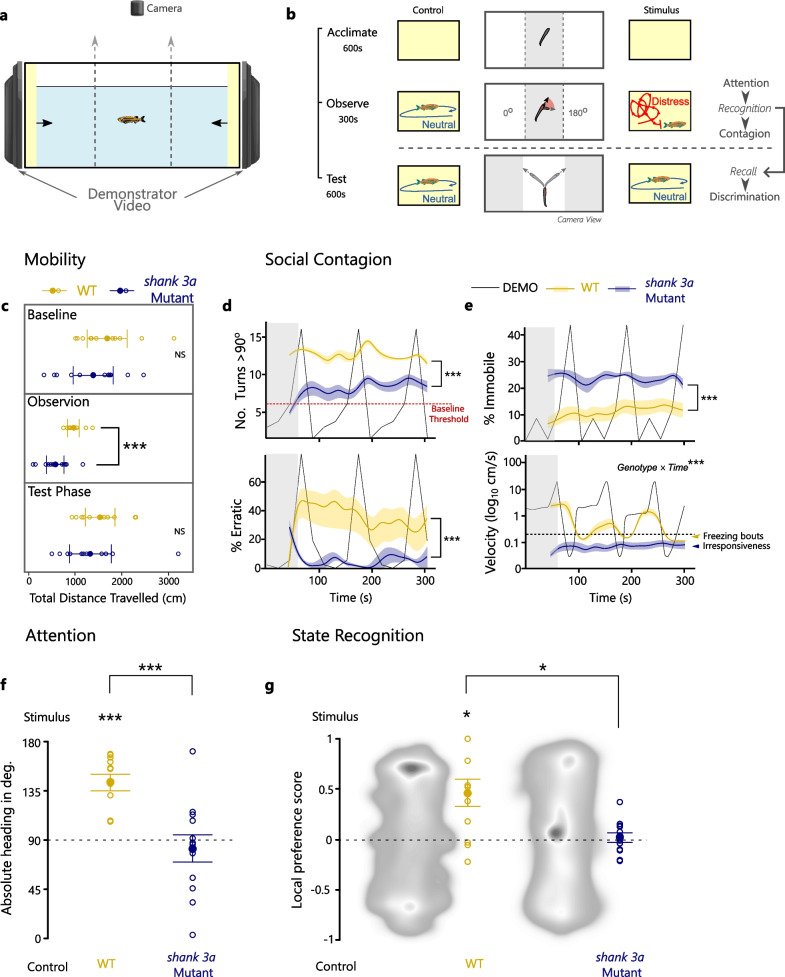
Experimental assessment of *shank3a* mutation effects on social contagion and underlying recognition and attention components. (**a**) The set-up included a corridor separated in three equal parts by removable transparent dividers, a camera with a birds-eye-view of the arena and monitors on either side of the tank for displaying demonstrator videos. (**b**) The experiment included three phases. Acclimation to the arena and background videos for 10 min, where baseline mobility during the last 5 min could be assessed. Observation of two contrasting videos from the central compartment for 5 min, where the demonstrator exhibited either neutral behaviour (control) or periodic distress (stimulus: erratic and freezing). Test of local preferences for 10 min, following removal of dividers and access to the whole tank, while both videos displayed the demonstrator in a neutral state. During the observation phase, fish mobility, orientation towards the distress behaviour (heading: 0–180°) and repetition of the observed erratic and freezing behaviour were measured. During the test phase, discrimination based on the local preference for either video was used to assess the recall of recognised differences between distress and neutral state. (**c**) Mobility in terms of total distance travelled, was lower in *Shank3a* mutants only during observation phase, suggesting no motor deficits are present. (**d**) Temporal changes in the directional changes exceeded baseline thresholds, and together with the proportion time erratic response was exhibited, following analogous behaviour in the stimulus video, were markedly lower in mutants, compared to wild-type animals. (**e**) Immobility, used to measure freezing, was greater in mutants, but this related to temporal differences in velocity that revealed an overall low activity in mutants compared to the freezing bouts in wild types. (**f**) Attention towards the distressed stimulus, compared to the neutral control, was greater in the wild-type animals but not in the *Shank3a* mutants. (**g**) Local preference scores revealed recognition deficits in the *Shank3a* mutants compared to wild types. Heat maps are representative examples with the least deviation from the mean. [*P ≤ 0.05, **P ≤ 0.01, ***P ≤ 0.001]

To test social fear contagion, we used a previously validated two-alternative video-playback approach [19]. The custom-built set-up includes a long corridor (arena: 14.5L, 29.5 × 14.5 × 11 cm) placed on top of an infra-red lightbox inside a dark cabinet, with two monitors on either side (Asus VG248, 1080 HD, 144 Hz rapid refresh rate) presenting focal fish with concurrent pre-recorded videos of a conspecific demonstrator, and with an overhanging camera for recording (Fig. 2a). The corridor is separated in three equal parts constructed by removable transparent dividers. This enables the use of a two-part protocol where focal animals are first acclimatised in the central compartment and allowed to observe and encode conflicting behavioural states presented between the two monitors and then given access to either monitor to test discrimination via local preferences (Fig. 2b).

During observation, the two playbacks exhibit the same demonstrator in two discrete states: a consistent neutral state (baseline swimming; control) on one monitor and an intermittent distress state (three bouts of erratic and freezing acts; affective stimulus) on the other. This allows the quantification of contagion in observers (erratic and freezing behaviour) and shifts in attention between observed neutral and distress states, while the use of the same demonstrator controlled for effects from individual differences that may be independent from behavioural state. During the observation phase animals are also able to collect information on the state presented on either monitor, which is required for recognition and quantified in the test phase. During the test phase, both monitors present the demonstrator in a neutral state, and thus cumulative local preferences can indicate discrimination dependent on the recall of previously recognised differences in observed behaviour (distress from neutral) rather than any effects from current differences in stimuli, e.g. differences in movement or in signals of imminent local danger.

### Demonstrators and video-playbacks

Playback videos were constructed by recording two naïve wild-type zebrafish unfamiliar and unrelated to the focal fish. Recordings were done in 1.5-L tanks, which were first video captured before the introduction of the demonstrators for use during the acclimatization phase of experiments. The tanks included an overhanging PVC tubing (diameter: 0.8 mm internal, 2.4 mm external) and obscured by their surroundings by opaque covers, but in view of a camera (goPro hero3 + , 60 fps, 1080 pixel resolution) kept in place outside one of the tanks’ glass walls, and behind an opaque acrylic sheet with a customised cut-out for the camera lens, that could be remotely operated to record behaviour. Demonstrators were individually kept in these tanks overnight and recorded the following day. During recording, we extracted a 200 s capture of the demonstrator in undisturbed baseline behaviour (neutral swimming) followed by the introduction of 0.75 ml of alarm substance from the overhanging tube and the recording of the behavioural response. The alarm substance used to elicit demonstrator response was extracted from live fish and appropriately stored using a previously reported protocol [19]. The response consisted of a stereotyped repertoire of erratic behaviour, i.e. fast zig-zagging movements, and freezing, characterised by immobility on the bottom of the tank with fast opercular movement.

Videos were then edited (VSDC© software; v. 6.3.6.18; Flash-Integro LLC, 2019) and included a 10-min playback of the demonstrator housing tank, used as background during acclimation; a 5-min video with the demonstrator exhibiting baseline swimming behaviour (neutral control), which included 3 repetitions of a 100 s recorded period; a 5-min video of the demonstrator periodically exhibiting distress, including 3 repetitions of a 60 s swimming period followed by a 40 s bout of the distress behaviour, i.e. erratic and freezing. During tests, videos were scaled on monitors to live-size proportions and focal fish were exposed to one of two playback replicates, one of a male and one of a female demonstrator, counterbalanced across individuals of either genotype (*shank3a* mutants or wild types). This controlled for demonstrator effects being either sex-specific or due to any individual phenotypic property.

### Behavioural test and recording protocols

In order to limit effects from prior social interactions, on the eve of experimental testing animals were moved to overnight individual housing in 1.5 L tanks kept at the same conditions as their original housing. On the day of testing animals were individually placed in the central division of the experimental arena and allowed to acclimatise for 10 min to playbacks of the empty demonstrator tank. Then, animals were kept in the central compartment and allowed to observe the two videos of the conflicting behavioural states (neutral and distress) for 5 min, with the side on which either state was presented being counterbalanced across experimental animals to control for side biases. After this period, videos shifted to the 10-min presentation of the neutral behaviour playback on both monitors, and the dividers delimiting the central compartment were lifted, allowing access to the whole arena (Fig. 2b). This enabled the quantification of cumulative local preferences towards either playback. Both the observation and test phase were recorded from above using via an infra-red sensitive camera (Henelec 300B; acquisition at 30 fps) for enhancing contrast against the infra-red light-box on the bottom of the tank. Videos were fed to a remote computer and recorded via the Pinnacle Studio software (v. 12, http://www.pinnaclesys.com). Individual recordings were then analyzed using the commercially video tracking software Ethovision XT© 11.0 (Noldus Inc., The Netherlands).

### Behavioural data extraction

From the recordings of each phase of the experiment, the movement of animals was automatically tracked and movement data (*x*, *y* coordinates) analysed to measure behaviour (Additional file 3: Data S1). Across experimental phases, we extracted overall mobility measures using the total distance travelled by animals in each period, including baseline mobility during the last 5 min of the acclimation period, as well as mobility during the observation and test phases. From movement data during observation, within the central compartment in which animals were restricted, attention to videos was measured by the absolute compass heading (*x* direction relative to the distress-stimulus video, ranging from 0° to 180°) and contagion was measured by the proportion time spent in erratic movement [acceleration > 8 cm/s^2^ and > 5 changes in direction/sec (> 90°)] and freezing (velocity < 0.2 cm/s), and directional change frequency (no. clockwise + no. anti-clockwise, > 90^O^) and velocity (cm/s) were additionally used to better qualify behavioural outputs. From the recordings of the test phase, during which access to the full tank was allowed, the third of the tank next to each video was set as regions of interest (ROIs) representing interaction zones and the cumulative time spent within each ROI was measured.

### RNA extraction and cDNA synthesis

For RNA extraction, a subsample of 9 fish per genotype were euthanised with an overdose of tricaine solution (MS222, Pharmaq; 500–1000 mg/L), the whole brain was collected directly into 500 µl lysis buffer (RNeasy Lipid Tissue Mini Kit-Qiagen) and stored at − 80^◦^C. Total RNA was extracted with the RNeasy Lipid Tissue Mini Kit (Qiagen) according to the manufacturer’s instructions, and the concentration, as well as the purity ratios (260 nm/280 nm and 260 nm/230 nm) estimated in the NanoDrop 2000 (Thermo Scientific). The same concentration of RNA for each sample was then reverse transcribed to cDNA (iScript cDNA Synthesis Kit, Biorad), following the manufacturer’s instructions. Briefly, a mix of nuclease-free water, 5 × iScript reaction mix (4 µl), iScript reverse transcriptase (1 µl), and RNA template were prepared in a 1.5 µl sterile tube in a final volume of 20 µl, and incubated in a PCR thermocycler in the following conditions: 5-min priming at 25 °C, 60-min reverse transcription at 42 °C, 5-min reverse transcription inactivation at 85 °C, and then and kept at 4 °C until tube collection. The samples were subsequently stored at -20 °C until further use.

### Gene expression

Quantitative real-time PCR (qPCR) was performed for the target genes (*bdnf*, *npas4, nlgn1, nlgn2, wnt3, neurod),* and the eukaryotic translation elongation *factor 1 alpha 1, like 1 (eef1a1l1*) was used as a reference gene [43]. Primers for the gene *Shank3a* were designed on Primer 3 (primer3.ut.ee), tested for quality in the FastPCR 5.4., and the PCR product was sent to sequence to confirm the amplicon size and the alignment with the target sequence. The qRT-PCR’s were performed in the Applied Biosystems quantstudio 7 thermocycler (7900 HT, Thermofisher) in 8 μl reactions, with SYBR Green PCR Master Mix (Applied Biosystems, Life Technologies), the primers at a concentration of 50 μM and the cDNA samples diluted 1:10. Thermocycling conditions were 5 min at 95° C, followed by 40 cycles: 95 °C for 30 s, specific annealing temperature for each primer for 30 s (Additional file 5: Table S1), and 72 °C. A melting curve was also included, with a program from 55 to 95 °C with 0.5° C increase changes and the presence of a single reaction product in each well was confirmed. All reactions were performed in triplicate, and the technical replicates were run on the same plate. To calculate the relative expression, the ΔΔCt method ( 2^–∆∆Ct^) was used (Additional file 4: Data S2), where Ct is the qPCR-generated cycle threshold and ΔCt is calculated by:

$$\Delta Ct = Ct_{{\left( {{\text{Target}}\,{\text{Gene}}} \right)}} - Ct_{{\left( {{\text{Reference}}\,{\text{Gene}}} \right)}}$$

and ΔΔCt by:

$$\Delta \Delta Ct = \Delta Ct_{{\left( {{\text{Target}}\,{\text{Sample}}} \right)}} - \Delta Ct_{{\left( {{\text{Reference}}\,{\text{Sample}}} \right)}}$$

### Analysis

Statistical analyses, calculations and graphical representations were carried out using the softwares Minitab® (v.17; Minitab Inc., State College, PA) and GraphPad© Prism (v.8.4.2; GraphPad Software LLC, San Diego, CA). The visualization of the genetic mutation was performed by the ApE© software (v2.0.5) and of the 3D protein structure by the open-source software ColabFold [45] that utilises MMseqs2 with AlphaFold2. Figures were edited and completed with illustrations using the software Adobe® Illustrator® (CS6, v.16.0.0; Adobe Systems Inc.) and Inkscape© (v. 1.2.2; Free Software Foundation Inc.).

From the behavioral test, the cumulative distance travelled by each animal (in cm) during the latter 5 min of the habituation period and during both the observation and test phase was compared between mutants and wildtypes using Welch’s 2-sample *t*-tests (due to unequal sample sizes). For the observation phase, we evaluated the social contagion of distress by measuring the percentage time exhibiting the behaviors observed in the distressed demonstrator, i.e. erratic and freezing (as per the defined kinematic thresholds), as well as average velocity for better screening of freezing bouts. These measures were compared between wild-type animals and *shank3a* mutants using a general linear model that included time bin, to test for temporal variations, and sex as added factors. Further, the number of directional turns exhibited by focal animals was compared to the maximum exhibited by demonstrators during their neutral swimming pattern to assess any motor deficits, using 1-sample Poisson rate tests, and compared between genotypes using 2-sample Poisson rate tests. Genotypic and temporal variations in average velocity were assessed via a general linear model for differentiating freezing bouts from irresponsiveness.

To characterise attention, we first tested whether mean absolute heading towards distress (0°–180°) significantly differed between genotype and sex using a general linear model. We then tested if it differed from divided orientation between distress and control playbacks (µ ≠ 90°) via 1-sample *t*-tests, both in wild-type animals and *shank3a* mutants. Finally, we quantified attentional differences by comparing mean absolute heading between wild-type animals and *shank3a* mutants using Welch’s 2-sample *t*-tests (due to unequal sample sizes).

To quantify recognition of the distress-state, we calculated individual local preference scores (*PS*) based on the time individuals spend in the ROI near the distressed-state stimulus (*T*_*S*_) compared to the control video (*T*_*C*_) using:

$$PS = \frac{{\left( {T_{s} - T_{c} } \right)}}{{\left( {T_{s} + T_{c} } \right)}}$$

where values range between -1 (full preference for control) and 1 (full preference for stimulus). To identify sex and genotype effects on *PS*, we used a general linear model. Then, discrimination ability in both wild types and mutants was ascertained by testing whether the mean *PS* for each group was significantly different form 0, using 1-sample *t*-tests. Comparisons between wild-type and mutant fish were performed using Welch’s 2-sample *t*-tests (due to unequal sample sizes).

For the characterization of *shank3* mutation effects on neuroplasticity, the relative expression of target genes was compared between a subsample of mutants and wild types by unpaired *t*-tests, with Welch’s approach for cases with unequal samples sizes (due to quality-control sample exclusions), and Mann–Whitney *U* tests when measures did not conform to normality. We then measured inter-correlations in expression levels between genetic markers, and performed correlation-based cluster analysis (complete linkage based on absolute correlation coefficients, |*r*|) followed by correlation-based principal components analysis (PCA), with varimax rotation (best for smaller sample sizes [46]), to identify groups of associated genes and extract separate neuroplasticity component scores relating to differentiated gene groups. Finally, multiple linear regression analyses were used to examine the effect of different neuroplasticity components to attentional control and state recognition measures.

## Results

### Social contagion effects and attention-recognition deficits

Baseline mobility, as estimated by the distance animals travelled in the arena, did not differ between *shank3a* mutants compared to wild-type animals during the last 5 min of the acclimation period (Welch’s: *t *_*20*_ = –1.06, *P* = 0.300, Cohen’s *d* = –0.442) or during the test phase (Welch’s: *t *_*19*_ = –0.82, *P* = 0.422, Cohen’s *d* = –0.342), and only during the observation phase (Welch’s: *t *_*18*_ = –3.78, *P* = 0.001, Cohen’s *d* = –1.578) were differences identified between genotypes (Fig. 2c).

During the observation phase, counts of directional changes (> 90^O^) exceeded baseline maxima (µ ≠ 11) set by demonstrators at neutral state in both wildtypes (*z* = *32.74, P* < 0.001) and mutants (*z* = 7.42*, P* < 0.001). However, both the rate of directional changes (rate _MUT_ = 7.35, rate _WT_ = 12.24; *z* = 14.41*, P* < 0.001) and their scaling via acceleration thresholds to erratic response (*R*^*2*^ = 0.216, *F*_*1,308*_ = 73.73, *P* < 0.001) were significantly lower in *shank3a* mutants compared to wild-type animals (Fig. 2d). Erratic response exhibited no significant sex effects (*R*^*2*^ = 0.001, *F*_*14,308*_ = 0.31, *P* = 0.580) or temporal variation (*R*^*2*^ = 0.025, *F*_*14,308*_ = 0.61, *P* = 0.858). Immobility was greater in *shank3a* mutants than wild types (*R*^*2*^ = 0.360, *F*_*1,308*_ = 142.78, *P* < 0.001; Fig. 2e), also without significant sex effects (*R*^*2*^ = 0.025, *F*_*14,308*_ = 0.87, *P* = 0.350) or temporal variation (*R*^*2*^ = 0.022, *F*_*14,308*_ = 0.67, *P* = 0.807). However, velocity differences between mutants and wild types were dependent on temporal changes (interaction: *R*^*2*^ = 0.270, *F*_*14,293*_ = 7.75, *P* < 0.001), where the constant immobility of mutants contrasted demonstrator-led periodic shifts between mobile states and freezing bouts in wild-type animals (Fig. 2e).

There were no sex effects on attention (*R*^*2*^ = 0.018, *F*_*1,22*_ = 0.59, *P* = 0.453; Additional file 1: Fig. S1), but genotype had strong and significant effects (*R*^*2*^ = 0.428, *F*_*1,22*_ = 15.48, *P* = 0.001). The differences in attention towards social stimuli between the two genotypes (Welch’s: *t *_*17*_ = 4.05, *P* = 0.001; Fig. 2f) were defined by contrasts between *shank3a* mutants exhibiting no significant difference from shared orientation between distress and neutral playbacks (*µ* = 82.7 ± 13 SE;* t* = –0.57, *P* = 0.579) and wild-type animals exhibiting orientation preferences towards the distress stimulus (*µ* = 142.99 ± 7.6 SE; *t* = 6.95, *P* < 0.001). Local preference during the post-observation test phase was also strongly predicted by genotype (*R*^*2*^ = 0.273, *F*_*1,22*_ = 8.30, *P* = 0.010), but was not affected by sex (*R*^*2*^ = 0.006, *F*_*1,22*_ = 0.20, *P* = 0.663; Additional file 1: Fig. S1). The differences in local-preference between the two genotypes (Welch’s: *t *_*12*_ = 2.71, *P* = 0.019; Fig. 2g) were a product of the *shank3a* mutants exhibiting no preferences (*PS: µ ≠ *0; *t* = 0.38, *P* = 0.711) in contrast to with wild types exhibiting significant preference for the playback where the demonstrator had been seen in distress (*PS: µ ≠ *0; *t* = 2.99, *P* = 0.014).

### Characterisation of neuroplasticity components

**Fig. 3:**
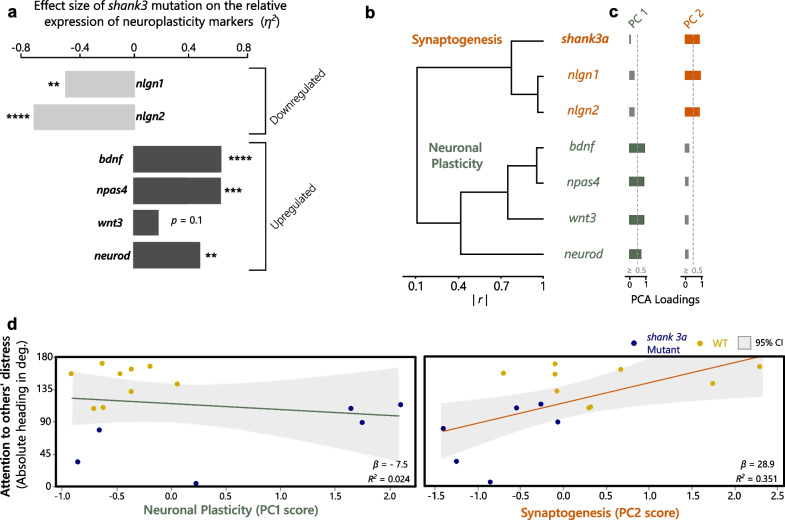
Quantification of changes in genetic neuroplasticity markers derived by the *shank3* mutation and analysis of their interrelated effects on attention. (**a**) Compared to wild types, *shank3a* mutants presented decreases in the expression of two neuroligin genes, *nlgn1* and *nlgn2*, and increases in the expression of the synaptic strengthening genes *npas4* and *bdnf*, and the neurogenesis genes *wnt3* and *neurod.* (**b**) Based on absolute correlations between their expression levels (|*r*|), neuroligins clustered with *shank3a*, which (**c**) was quantified by PCA as a functionality-based composite synaptogenesis component, while all other genes clustered separately and quantified as a broad neuronal plasticity component. (**d**) Only the synaptogenesis component presented significant associations to the levels of attention towards the distressed conspecific exhibited during the social contagion test. [*P ≤ 0.05, **P ≤ 0.01, ***P ≤ 0.001, ****P ≤ 0.0001]

Comparisons in whole-brain gene expression between *shank3* mutants and wild-type fish, revealed a strong and significant downregulation of neuroligins (*nlgn1: t*_15_ = 3.92, *P* = 0.0014*; nlgn2: t*_13_ = 5.96, *P* < 0.0001), a significant upregulation of *bdnf* (*t*_16_ = 5.26, *P* < 0.0001), *npas4* (*U*_15_ = 2, *P* = 0.0003) and *neurod* (*t*_16_ = 3.86, *P* = 0.0014) expression, and a small non-significant upregulation of *wnt3* (*t*_16_ = 1.78, *P* = 0.0954) expression (Additional file 2: Fig. S2; Fig. 3a). Downregulated and upregulated genes had different patterns of co-expression, with downregulated neuroligins clustering with *shank3* expression levels and all other genes clustering separately (Fig. 3b). Consistently, the PCA analysis (KMO = 0.6, Bartlett’s: χ^2^ = 95.51, p < 0.001) extracted 2 principal components (PC, eigenvalue > 1): PC1 presenting significant loadings (> 0.5) of different neuronal plasticity genes, involved in synaptic strengthening (*bdnf*, *npas4)*, neuronal proliferation (*wnt3)* and neuronal differentiation (*neurod)*; and PC2 presenting significant loadings of synaptogenesis genes (*nlgn1, nlgn2*) and *shank3* (Fig. 3c)*.* Together, the neuroplasticity components had a strong combined effect on absolute heading towards the distressed conspecific (model: *R*^*2*^ = *0.375*), but the general neuronal plasticity component had no significant effect (PC1: *R*^*2*^ = *0.024, F*_*1,14*_ = 0.46, *P* = 0.511) and only the synaptogenesis component had a strong positive influence on attention (PC2*: R*^*2*^ = *0.351, F*_*1,14*_ = 6.73, *P* = 0.023; Fig. 3d). Conversely, the much weaker combined effect on recognition (PS, model: *R*^*2*^ = *0.156*) exhibited no significant contributions from either the general neuronal plasticity component (PC1: *R*^*2*^ = *0.068, F*_*1,14*_ = 0.97, *P* = 0.344) or the synaptogenesis component (PC2: *R*^*2*^ = *0.087, F*_*1,14*_ = 1.24, *P* = 0.287).

## Discussion

The global prevalence of ASD has been exhibiting an ongoing increase, which has imposed an urgency to the development of effective animal models that enable the precise targeting of candidate mechanisms [47, 48]. The consensus is that genetic animal models can identify risk factors, hereditary components and neurodevelopmental mechanisms, which can contribute to better detection, treatment and prevention, for which the utility of zebrafish extents to fast screening throughput, genetic tractability and well-developed, automated phenotyping tools [48–50]. So far, this utility of zebrafish has been limited to the identification of simple phenotypic effects of genetically modulated ASD-like pathology, such as reduced tendencies to interact with others, aggression, or deficits in group cohesion and integration [49, 50]. Here we expand this and demonstrate the potential of zebrafish for the development of genetic models that target compound social ASD phenotypes, such as the contribution of the autism candidate gene *shank3* to attention-based deficits in the social recognition and transmission of affective behaviour. Notably, such complex phenotypes, and the characterisation of their different cognitive components, has seldom been described in any ASD model outside primates, as revealed in systematic revisions of the literature [48]. Thus, the development of nuanced behavioural paradigms is crucial for capturing the full effects of candidate ASD mechanisms.

Individuals with autism can exhibit a diverse set of phenotypes with varying functional capacities, but challenges in social interactions tend to be particularly widespread among all forms of ASD [51, 52]. One of the most typical contributors to this is difficulties with verbal and non-verbal communication [51]. All forms of communication involve the ability of receivers to perceive the signals of senders, a process that provides bonding and co-operation benefits to social living and which can extend to the communication of affective states [53, 54]. Autistic individuals can present deficits in this ability to various degrees, but these deficits can be marked even in those with Asperger’s syndrome and so-called high-functioning autistic individuals, which have lower delays in cognitive development [55]. These deficits extent to difficulties to perceive expressions of affective states [56], which is expected to lead to the decreased capacity of their transmission, i.e. social contagion. However, the underlying genetic mechanisms are unknown.

In our study, we found that mutation to the zebrafish *shank3a* gene, the isoform considered to show the greatest orthology to the human gene and to transcribe the most conserved SHANK3 protein [21, 22], elicits deficits in contagion. When wild-type animals were presented with erratic behaviour in distressed demonstrators, they also exhibited consistently higher levels of erratic behaviour, but in *shank3a* mutants this phenotype was markedly decreased (Fig. 2d). In contrast, the time spent immobile was much greater in *shank3a* mutants throughout exposure to the distressed demonstrators, which periodically expressed freezing bouts (Fig. 2e). On the surface, this may appear as if mutants presented motor deficits, but neither baseline mobility nor mobility during the test phase were different between genotypes (Fig. 2c). In lieu of motor differences, an alternative explanation could be that mutants were responding with greater freezing contagion than wild types. However, in contrast to wild types, that exhibited velocity changes corresponding to shifts between movement and freezing, analogous to those observed in distressed demonstrators, mutants remained with low mobility throughout the observation phase (Fig. 2e). This suggests that neither the saliency of distress cues, such as high mobility during erratic bouts and sudden shifts to freezing, nor the contrast of this behaviour against the neutral behaviour demonstrated in the opposite side elicited any response in mutants. The saliency of cues and the implication of changes in motion remains an open question for the contributors to deficits in affective state recognition observed in humans with ASD [6], and here we get a first indication that global disruptions to synaptic function imposed by the *shank3* mutation may drive this via attentional or recognition impairments.

Social contagion relies on perception–action mechanisms that enable individuals to repeat the behaviour observed in others. According to evolutionary accounts, these mechanisms can scale up to affect-sharing and ultimately empathic concern [4], yet their involvement is not limited to the sharing of internal states but also to simpler behavioural mimicry phenotypes. The difference is specific to the recognition component, where a behavioural expression needs to be recognised as an internal state for that state to be shared, otherwise its replication is simply motor imitation. In zebrafish, distress behaviour is expressed in response to imminent danger, such as the presence of a predator, or to signals of danger originating in others, such as the release of alarm substance following injury or the expression of erratic movements or freezing. The replication of this behaviour alone can provide threat-mitigation functions that are independent of recognising the underlying distress of others. However, as we previously demonstrated, zebrafish can discriminate between individuals that previously were observed to be distressed from those remaining in a neutral state, and that this is modulated by oxytocin and its regulation of excitatory and inhibitory signalling [19]. Both these types of signalling can be impaired by the synaptic defects imposed by *shank3* mutations [24]. Here we find that the ability to encode differences between distress and neutral behaviour during observation, and to later use these to organise local preferences towards others seen to be distressed, is severely impaired in *shank3* mutants (Fig. 2g). Indeed, contrary to oxytocin mutant zebrafish that simply reverse their preference for the demonstrator that remained neutral [19], *shank3* mutants show no preference. This suggests that the pathology of these mutants is not on the motivation to approach, but on the overall inability to discriminate behavioural differences between the affective distress repertoire and the neutral state. This adds further support that contagion deficits may derive from attentional impairments.

In humans, social contagion is rarely quantified for assessing the ability to communicate affective states and instead the focus has been on trait and state empathic components, typically assessed by questionnaires, and via facial-expression recognition tasks [9]. This comes down to deficiencies in discriminating salient affective expressions, such as fear or distress, from neutral ones [6]. However, the evidence is often unclear on whether these ASD-imposed deficiencies are due to difficulties in processing behavioural cues from others or due to paying attention to them. In some rare examples that attention is more explicitly measured via gaze and eye movements, the evidence remains divided, with some demonstrating that attentional control underlies ASD recognition deficits [32, 33] and others showing that recognition deficits in higher-functioning autistic individuals are independent from attentional difficulties [35]. In the case of *shank3* derived ASD pathologies, this has yet to be assessed. Here, we were able to extract measures of attention by looking at the orientation of individuals towards the distressed demonstrator stimulus compared to the control. We previously demonstrated that attention to distress behaviour is not simply elicited by the saliency of motion, because individuals shift their attention equally towards highly mobile erratic acts and highly immobile freezing behaviour in others [19]. This translates to average preferences in orientation towards distressed compared to neutral behaviour, which was also clearly exhibited by wild-type animals in this study (Fig. 2f). In contrast, *shank3a* mutants did not shift attention towards either the distress or the neutral behaviour. Together with the lacking response seen in the contagion measures, this reveals an overall attentional deficit towards salient affective behaviour in others.

Although genetically derived SHANK3 impairments have been associated to ASD pathology in general, their implication in the Phelan-McDermid syndrome, as well as evidence of its functional effects on connectivity patterns in the brain, similarly suggest that attentional deficits may be the most parsimonious explanation for some SHANK3-related ASD phenotypes in humans. In particular, the neurodevelopmental issues imposed by the Phelan-McDermid extent to severe attentional deficits that relate to the SHANK3 protein’s control over region-specific and shared interactome patterns in the brain [57]. Moreover, mutation to the *shank3* gene in primates induces global decreases in the strength of functional connectivity, while localised connectivity strength increases in some regions and decreases in others, all of which contribute to delayed visual reflexes and low attentional control, despite no apparent deficiencies in recognising facial expressions of threat [58]. This provides some comparative value to our findings here, against the limited evidence in humans and other model species.

Added insight is provided by our characterization of neuroplasticity, which provides evidence on how *shank3a* mutation in zebrafish modulates the attentional deficits we describe. On the one hand, the expression of neuroligin genes (*nlgn1, nlgn2*) was downregulated in the *shank3a* mutants (Fig. 3a), which is consistent with the well-described postsynaptic binding interaction between SHANK3 and neuroligins [27, 36], and with evidence of postsynaptic neuroligin decreases in *shank3* mutant rats [37]. On the other hand, the *shank3* mutation drives a strong upregulation of a neuronal differentiation gene, *neurod*, and two immediate early genes involved in synaptic strengthening, *bdnf* and *npas4* (Fig. 3a). Increases in *neurod* RNA expression have also been documented in rat autism models and although this induced neuronal differentiation, it also suppressed differentiation to macroglial subtypes [41]; namely astrocytes and oligodentrites, which could extent the negative neurotransmission and synaptogenesis effects of the *shank3*-neuroligin downregulation [59, 60]. In turn, expression of *npas4* in mice regulates inhibitory synapse redistribution, by targeting *bdnf* [40], and in zebrafish the expression of both these genes is modulated by social interactions [43]. Thus, changes in the expression of these genes are likely an effect specific to inhibitory neurons and their upregulated expression in mutants might present a case of functional compensation in this socio-behavioural context. Indeed, in mice, autism-related genetic deficits in social interactions were recovered by *npas4* injections to the prefrontal cortex [38], and contrasting autism-induced changes in expression between *shank3* and *bdnf* were recently demonstrated in rat autism models, where hippocampal and cerebellar increases in *shank3* expression were paralleled with decreases in *bdnf* expression [39]. Nevertheless, the expression levels of all upregulated genes in zebrafish mutants presented correlational clustering whose composite component scores (Fig. 3b, c) predicted neither of the two cognitive processes identified here as implicit to the social contagion deficits in *shank3* mutants, that is attentional control and distress recognition. Conversely, the corresponding component score derived from the separate synaptogenesis cluster of the correlated expression of neuroligins and *shank3a* (Fig. 3b, c) positively predicted the levels of attention individuals exhibited towards the distressed conspecific (Fig. 3d). This indicates a potential functional mechanism at the neuronal connection level for the attentional deficits in *shank3* mutants, which fits expectations for the shank-3 neuroligin-neurexin coupled functional network suggested to drive similar effects in humans and mammalian models [27, 36, 37].

## Limitations

For multifaceted disorders like ASD, it is difficult or even impossible to develop animal models that capture all aspects of its social pathology. Although a valuable tool for disentangling simpler phenotypes and identifying specific mechanisms, including the role of genetic determinants, animals still present evolutionarily distinct backgrounds and adaptations that are not full representatives of human biology. The question of ‘better’ model organisms is misleading given the phenotypes in question; their fast-screening throughput, their putative mechanisms and their development need to be examined before modelling any specific research question. Thus, complementing existing mammalian ASD models with zebrafish may rely on aspects of their social behaviour that are better representatives of specific target phenotypes, such as their tendency to form groups, from smaller social structures to larger collectives, which represents a human phenotype that established rodent models cannot [49, 50]. This also includes the sharing of social affective information, such as in the case of social contagion, which in rodents is usually limited to dyads and dependent on familiarity, kinship or bonding [3, 4, 14]. However, as the phenotype presented here reveals, our understanding of zebrafish social phenotypes and their genetic mechanisms is relatively limited and needs better development for enabling the use of this model with an improved translational potential. In addition, some cognitive components or socio-environmental conditions related to human social life that can shape genetic predispositions to ASD deficits in these phenotypes cannot be fully captured in zebrafish, for example cultural aspects, the influence of language, and aspects of mentalizing and consciousness that are specific to cortical functions and elicit complex empathic phenotypes evolved with these functions in humans [4]. Other open questions for modelling social health in zebrafish include the characterisation of individualities and personality-like differences, and their neuroendocrinology, as well as characterising social homeostasis and loneliness phenotypes, which together call for further translational work with this model [61].

In terms of modelling potential genetic mechanisms related to ASD-like phenotypes, zebrafish present some translational complications due to the teleost genome duplication event that resulted in the two paralogues we find in *shank3* and other genes, whereas humans and other mammals have only one copy [50]. Although the paralogue we selected to target here was one considered to be more representative for modelling the human SHANK3 protein functions [21, 22], genetic differences resulting from the genomic duplication can complicate the interpretation of phenotypic effects, particularly if those effects are moderated by an intact paralogous gene. In turn, for genes that have functional effects on brain physiology, like the synaptic plasticity implications of SHANK3, limitations extent to differences in brain development, anatomy and a lacking methodology for imaging/visualising and targeting neural systems more precisely, such as in more established rodent models [62, 63]. Finally, while the fast zebrafish social development and phenotyping throughput can address issues with large-scale pharmacological screens in mammals [64], their use for identifying genetic risk factors can be complicated due to difficulties in capturing pathology and risk changes. In particular, pathology in zebrafish is approximated by similarities in phenotype and the direction of genetic effects, such as the reduced attention and recognition identified here. However, in contrast to the systematically reviewed thresholds in composite phenotypes for identifying ASD in humans [51, 52], in zebrafish and most other models this remains difficult. Therefore, better population-based approaches in addressing this in model species are necessary for developing translational tools for detection, treatment, and prevention.

It is also important to note that in this study we have demonstrated that *shank3* is necessary for the expression of social contagion but, because we did not run a gain of function experiment, we have not shown it is sufficient for the expression of the target phenotype. Furthermore, we cannot ensure that the expression of neuroplasticity genes faithfully represents the mechanistic similarities identified in humans or mammalian models, nor can we be certain with the quantitative resolution described herein of either their localised or cellular-level effects, nor of their effects in terms of broader connectivity patterns.

## Conclusions

The association of ASD pathology to specific cognitive components and leading genetic mechanisms is hard to ascertain without controlled behavioural paradigms and the precise targeting of candidate genes. Following amassing support for the use of animal models, here we address these issues by the development of a zebrafish model that targets a specific candidate gene and that enables its association to the cognitive component that drives social contagion deficits: the impaired attention to salient behavioural cues that prohibits the recognition of underlying affective states in others. Thus, in addressing the impaired communication of affective states in ASD pathologies it is important to examine not only deficits in the transmission of signals, but also whether these deficits rely on attention or recognition. SHANK3 is a protein that plays a prominent role in synapse functioning and plasticity, a requisite for the development of functional connectivity patters implicated in attention [57, 58]. With our current findings, we conclude that attentional components to affective communication deficits in ASD individuals should expand focus on contributions from the *shank3* genetic mutation, as well as its functional effects on neuroplasticity and particularly its neuroligin-coupled modulation of synaptic formation.

## Supplementary Information

**Additional file 1**. Figures showing sex comparisons in attention and recognition measures for both wild-types and mutants.

**Additional file 2**. Figures showing differences in expression levels between wild-types and mutants for each neuroplasticity marker.

**Additional file 3**. Source data used for behavioural comparisons.

**Additional file 4.** Source data used for neuroplastcity characterisation.

**Additional file 5.** Primer sequences and parameters used for the qRT-PCR analyses.

## Data Availability

All data generated or analysed during this study are included in this published article and its supplementary information files.
