# Supplementary material for: Autism-associated gene shank3 is necessary for social contagion in zebrafish
Source: Mol Autism. 2023 Jun 30;14:23. doi: 10.1186/s13229-023-00555-4 (PMC10311831; doi:10.1186/s13229-023-00555-4)
Supplement: Supplementary file 1 — Additional file 1. Figures showing sex comparisons in attention and recognition measures for both wild-types and mutants. [file 13229_2023_555_MOESM1_ESM.pdf]

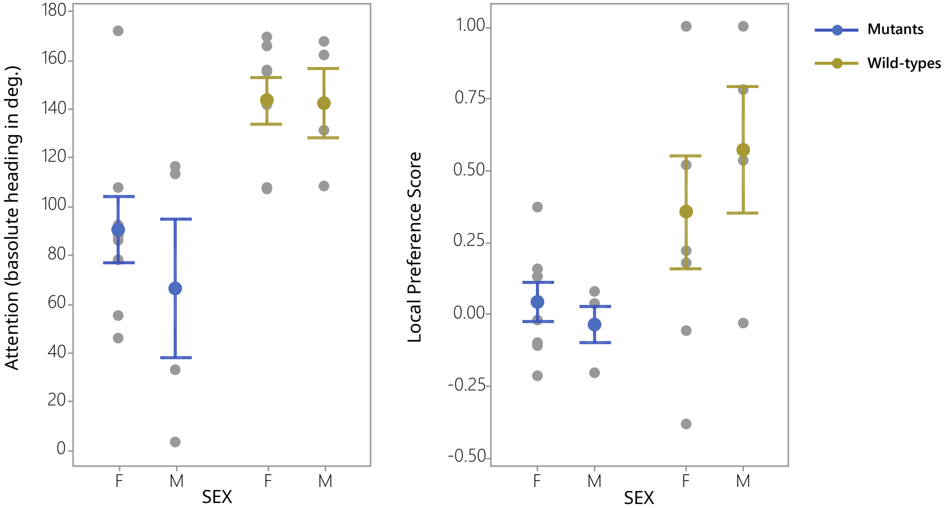

Figure S1: Comparisons of males (M) to females (F) of *shank3a* mutants and their wild-type siblings in attention towards distress during observation and local preference at the test phase of the behavioural experiment
