# Supplementary material for: Autism-associated gene shank3 is necessary for social contagion in zebrafish
Source: Mol Autism. 2023 Jun 30;14:23. doi: 10.1186/s13229-023-00555-4 (PMC10311831; doi:10.1186/s13229-023-00555-4)
Supplement: Supplementary file 2 — Additional file 2. Figures showing differences in expression levels between wild-types and mutants for each neuroplasticity marker. [file 13229_2023_555_MOESM2_ESM.pdf]

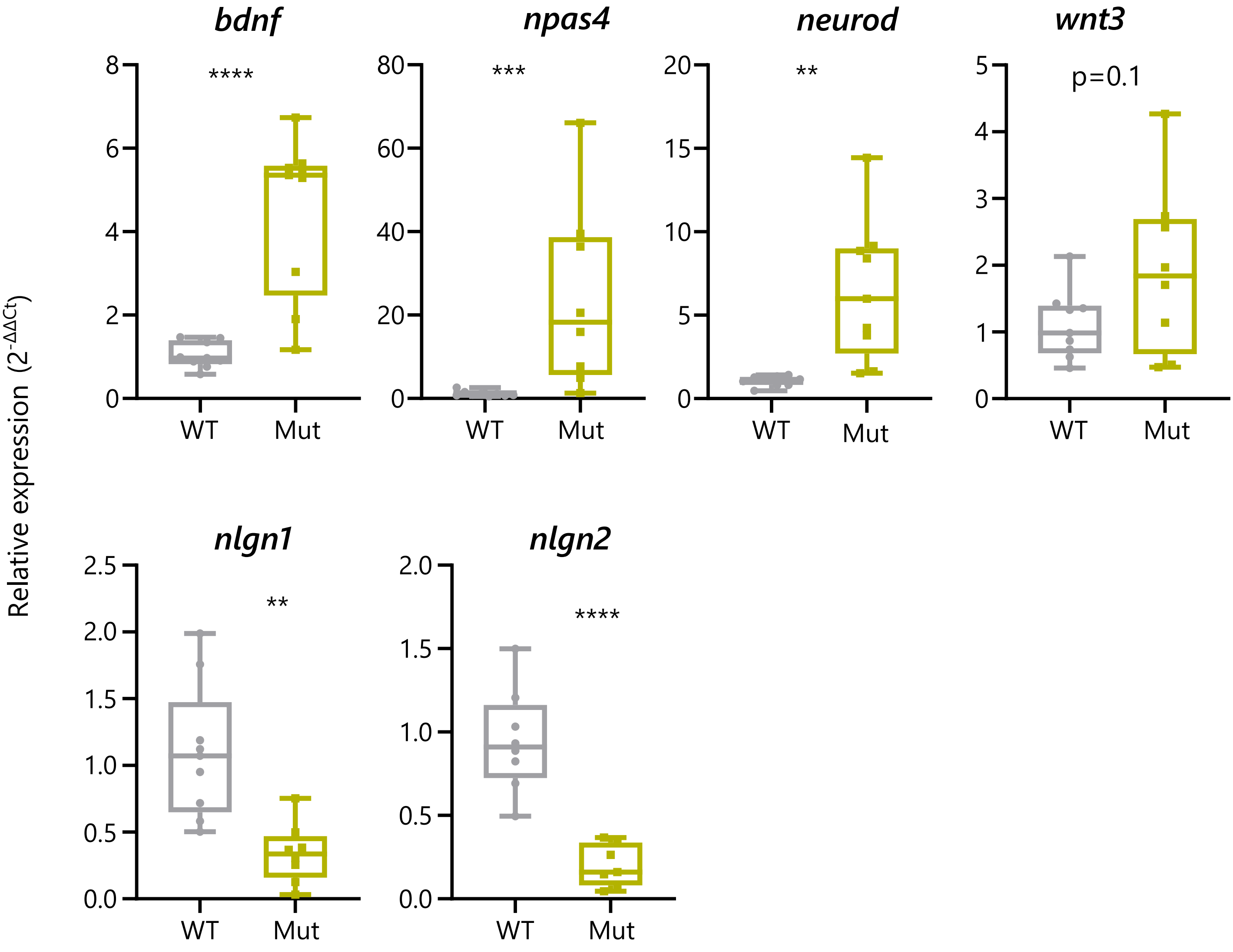

Figure S2: Comparisons between shank3a mutants (Mut) and their wild-type siblings in relative expression of RNA levels facross selected neuroplasticity genes.
