# Supplementary material for: Autism-associated gene shank3 is necessary for social contagion in zebrafish
Source: Mol Autism. 2023 Jun 30;14:23. doi: 10.1186/s13229-023-00555-4 (PMC10311831; doi:10.1186/s13229-023-00555-4)
Supplement: Supplementary file 5 — Additional file 5. Primer sequences and parameters used for the qRT-PCR analyses. [file 13229_2023_555_MOESM5_ESM.docx]

| **Table S1:** Primer sequences and qRT-PCR parameters | | | |  |
| --- | --- | --- | --- | --- |
| **Abbreviation** | **Accession No.** | **Primer Sequences (5’ → 3’)** | **Annealing temperature (°C)** | **Amplicon length (bp)** |
| *Sank3a* | XM_009293439.3 | F-TCCAGCATGTCCACACTGTC | 60 | 134 |
|  |  | R- TTGCTGAGCTGGGACTTGAG |  |  |
| *bdnf* | NM_131595 | F- GCTGCCGAGGAATAGACAAG | 58 | 157 |
|  |  | R- CTGCCCCTCTTAATGGTCAA |  |  |
| *npas4a* | NM_001045321 | F- GACACGGGTTGAGAATGGTT | 59 | 165 |
|  |  | R- GCACCAAGCACCCTGTAAAT |  |  |
| *nlgn1* | NM_001142265 | F- TCAACGAGGTCAGCCAGATA | 59 | 221 |
|  |  | R- TGAAGCACCGACAGCAATAG |  |  |
| *nlgn2a/ nlgn2b* | NM_001166336/ NM_001166329 | F- GTCTGCCAAAGGGAACTATG | 59 | 157 |
|  |  | R- ATGGTGGGACAGGATGAGTA |  |  |
| *wnt3* | NM_001114552 | F- CTGTTGGGGGACTACCTGAA | 57 | 108 |
|  |  | R- GGCGTATTTGGCTCGTAGTG |  |  |
| *neurod1* | NM_130978 | F- AAGTCAGATCCCTGCGTCAT | 63 | 185 |
|  |  | R- GGGAATTGTGCAACTCTGC |  |  |
| *eef1a1l1* | NM_131263.1 | F- GCTTCTCTACCTACCCTCCTCT | 60 | 97 |
|  |  | R- CCGATTTTCTTCTCAACGCTCT |  |  |
| F- Forward | R- Reverse |  |  |  |
